# Tautomerism in 10-(hy­droxy­imino)­phenanthren-9-one

**DOI:** 10.1107/S160053681203749X

**Published:** 2012-09-08

**Authors:** Dinesh G. Patel, Jason B. Benedict

**Affiliations:** a Department of Chemistry, University at Buffalo, Buffalo, NY 14260-3000, USA

## Abstract

In the title compound, C_14_H_9_NO_2_, a static disorder exists between the keto–oxime and hy­droxy–nitroso tautomers, in an approximate ratio of 4.6:1, based on refined occupancies for disordered parts. No inter­molecular hydrogen bonding is present in the crystal structure. Instead, both tautomers exhibit similar intra­molecular O—H⋯O hydrogen bonds.

## Related literature

For information on tautomerization in *ortho*-hy­droxy­nitroso aromatic compounds, see: Enchev *et al.* (2003 ▶); Terent’ev & Stankyavichyus (1988 ▶). For the role of *ortho*-hy­droxy­nitroso aromatic compounds in metal complexation and in photochromic spiro­oxazines, see: Barjesteh *et al.* (1996 ▶); Patel *et al.* (2005 ▶, 2010 ▶). For the spectrochemical characterization of the title compound, see: Kumar *et al.* (2009 ▶).
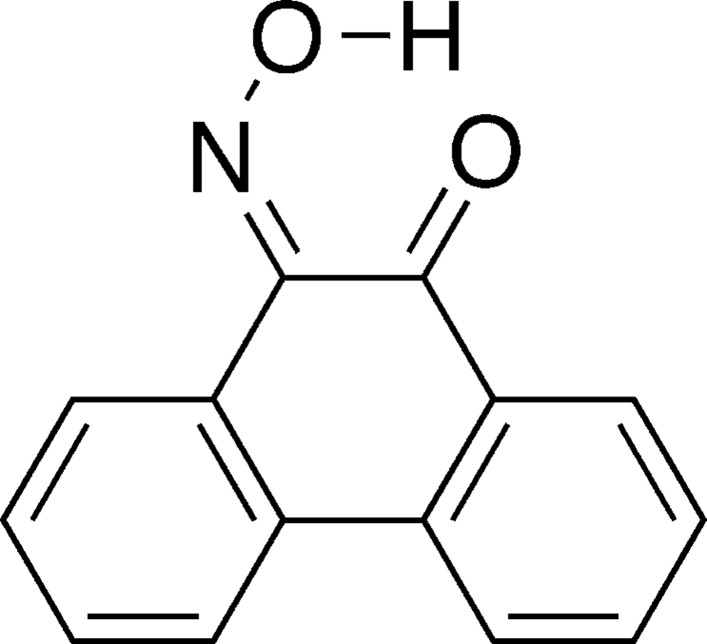


## Experimental

### 

#### Crystal data

C_14_H_9_NO_2_
*M* *_r_* = 223.22
Orthorhombic, 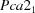

*a* = 17.4505 (15) Å
*b* = 3.7875 (3) Å
*c* = 15.1669 (13) Å
*V* = 1002.44 (15) Å^3^
*Z* = 4
Mo *K*α radiation
μ = 0.10 mm^−1^
*T* = 90 K
0.24 × 0.10 × 0.06 mm

#### Data collection

Bruker APEXII CCD diffractometer
Absorption correction: multi-scan (*SADABS*; Bruker, 2008 ▶) *T*_min_ = 0.976, *T*_max_ = 0.994
16616 measured reflections
1068 independent reflections
989 reflections with *I* > 2σ(*I*)
*R*_int_ = 0.032

#### Refinement

*R*[*F* ^2^ > 2σ(*F* ^2^)] = 0.036
*wR*(*F* ^2^) = 0.099
*S* = 1.07
1068 reflections
166 parameters
2 restraints
H-atom parameters constrained
Δρ_max_ = 0.21 e Å^−3^
Δρ_min_ = −0.15 e Å^−3^

### 

Data collection: *APEX2* (Bruker, 2008 ▶); cell refinement: *SAINT* (Bruker, 2008 ▶); data reduction: *SAINT* and *XPREP* (Bruker, 2008 ▶); program(s) used to solve structure: *SHELXS97* (Sheldrick, 2008 ▶); program(s) used to refine structure: *SHELXL97* (Sheldrick, 2008 ▶); molecular graphics: *OLEX2* (Dolomanov *et al.*, 2009 ▶); software used to prepare material for publication: *OLEX2*.

## Supplementary Material

Crystal structure: contains datablock(s) I, global. DOI: 10.1107/S160053681203749X/bh2448sup1.cif

Structure factors: contains datablock(s) I. DOI: 10.1107/S160053681203749X/bh2448Isup2.hkl

Supplementary material file. DOI: 10.1107/S160053681203749X/bh2448Isup3.cml

Additional supplementary materials:  crystallographic information; 3D view; checkCIF report

## Figures and Tables

**Table 1:** Hydrogen-bond geometry (Å, °)

*D*—H⋯*A*	*D*—H	H⋯*A*	*D*⋯*A*	*D*—H⋯*A*
O2—H2⋯O1	0.84	1.76	2.499 (8)	146
O1*A*—H1*A*⋯O2*A*	0.84	1.93	2.54 (2)	128

**Fig. 1.**
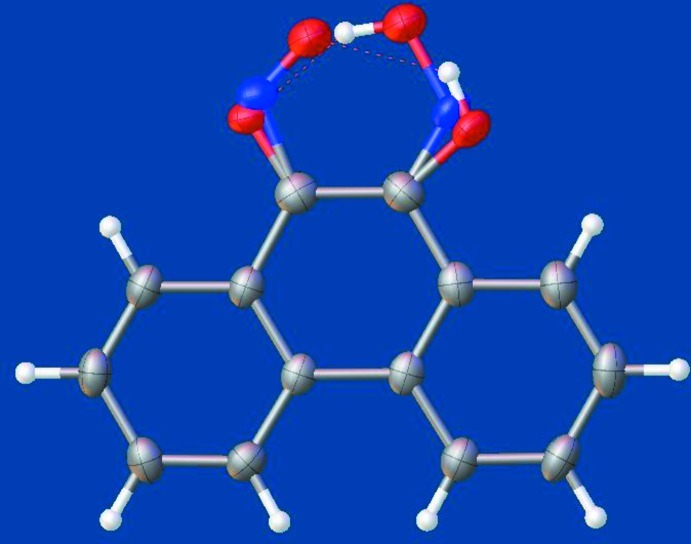
ORTEP view (Dolomanov et al., 2009) of the title molecule. Dashed lines represent intramolecular hydrogen bonds.

**Fig. 2.**
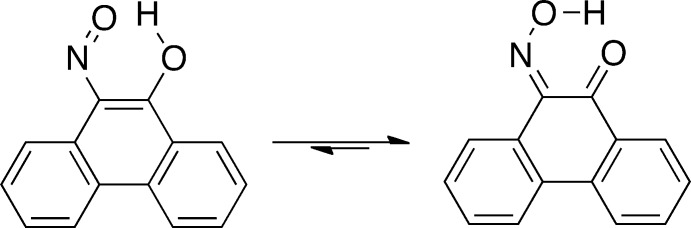
The tautomerism in the title compound.

## supplementary crystallographic information

### Comment

The title compound (Fig. 1) is an *ortho*-hydroxynitroso aromatic compound, which may have a role in metal complexation and in photochromic spirooxazines (Barjesteh *et al.*, 1996; Patel *et al.*, 2005, 2010). A tautomeric disorder was observed in this structure (Enchev *et al.*, 2003; Terent'ev & Stankyavichyus, 1988). Without accounting for the disorder, a Q-peak of approximately 0.9 electron/Å^3^ was located at the position of O2A in the disorder model. Prior to modeling the disorder, *R*_1_ residual was approximately 0.06. With the chemically sensible disorder model, the final residual is *R*_1_ = 0.0361, justifying the reduced number of parameters and minimal *EADP* constraints and *FLAT* restraint (see *Experimental*). The final refinement indicated the two tautomers are present in an 0.827 (3) to 0.173 (3) ratio.

### Experimental

The title compound was synthesized according to a previously published procedure (Terent'ev & Stankyavichyus, 1988). Briefly, commercially available phenanthrene-9,10-quinone (1.500 g, 7.20 mmol) was refluxed in ethanol (100 ml) and chloroform (20 ml). To this solution was added hydroxylamine hydrochloride (0.500 g, 7.20 mmol dissolved in 20 ml of water) dropwise. After refluxing overnight, solvent was removed and the resulting orange solid was recrystallized from aqueous ethanol (1.086 g, 68%). Spectral characterization matched that in the literature (Kumar *et al.*, 2009).

### Refinement

All H atoms were initially located in a difference Fourier map and were refined with a riding model. H atoms were placed in geometrically idealized positions and constrained to ride on their parent atoms with C—H distances fixed to 0.95 Å and O—H distances fixed to 0.84 Å. *U*_iso_ values were fixed as *U*_iso_(H) = 1.2*U*_eq_(parent C) and *U*_iso_(H) = 1.5*U*_eq_(parent O). Atoms N1A, O2A, H1A and O1A were restrained to be coplanar, and the same anisotropic displacement parameters were used for pairs of disordered atoms N1/O1A, O2/O2A and N1A/O1. Measured Friedel pairs (983) were merged in the final refinement.

### Crystal data

**Table:**

C_14_H_9_NO_2_	*F*(000) = 464
*M**_r_* = 223.22	*D*_x_ = 1.479 Mg m^−^^3^
Orthorhombic, *P**c**a*2_1_	Mo *K*α radiation, λ = 0.71073 Å
Hall symbol: P 2c -2ac	Cell parameters from 250 reflections
*a* = 17.4505 (15) Å	θ = 2.3–26.0°
*b* = 3.7875 (3) Å	µ = 0.10 mm^−^^1^
*c* = 15.1669 (13) Å	*T* = 90 K
*V* = 1002.44 (15) Å^3^	Plate, yellow
*Z* = 4	0.24 × 0.10 × 0.06 mm

### Data collection

**Table:**

Bruker APEXII CCD diffractometer	1068 independent reflections
Radiation source: rotating anode	989 reflections with *I* > 2σ(*I*)
Optics monochromator	*R*_int_ = 0.032
w scans	θ_max_ = 26.4°, θ_min_ = 2.3°
Absorption correction: multi-scan (*SADABS*; Bruker, 2008)	*h* = −21→21
*T*_min_ = 0.976, *T*_max_ = 0.994	*k* = −4→4
16616 measured reflections	*l* = −18→18

### Refinement

**Table:**

Refinement on *F*^2^	Primary atom site location: structure-invariant direct methods
Least-squares matrix: full	Secondary atom site location: difference Fourier map
*R*[*F*^2^ > 2σ(*F*^2^)] = 0.036	Hydrogen site location: inferred from neighbouring sites
*wR*(*F*^2^) = 0.099	H-atom parameters constrained
*S* = 1.07	*w* = 1/[σ^2^(*F*_o_^2^) + (0.0547*P*)^2^ + 0.3852*P*] where *P* = (*F*_o_^2^ + 2*F*_c_^2^)/3
1068 reflections	(Δ/σ)_max_ < 0.001
166 parameters	Δρ_max_ = 0.21 e Å^−^^3^
2 restraints	Δρ_min_ = −0.15 e Å^−^^3^
18 constraints	

### Special details

**Table:**

Experimental. Data were collected with five ω scans in 0.5° increments with 20 s. exposures per degree. Crystal-to-detector distance was 40 mm. 18620 full and partial reflections were integrated.

### Fractional atomic coordinates and isotropic or equivalent isotropic displacement parameters (Å^2^)

**Table:**

	*x*	*y*	*z*	*U*_iso_*/*U*_eq_	Occ. (<1)
O1	0.7027 (5)	0.491 (3)	0.5919 (3)	0.0366 (12)	0.827 (5)
O1A	0.5549 (10)	0.199 (7)	0.6677 (16)	0.0362 (9)	0.173 (5)
H1A	0.5911	0.2311	0.7033	0.054*	0.173 (5)
O2	0.63991 (15)	0.2459 (9)	0.72678 (18)	0.0500 (8)	0.827 (5)
H2	0.6762	0.3150	0.6947	0.075*	0.827 (5)
O2A	0.6923 (8)	0.414 (5)	0.6788 (9)	0.0500 (8)	0.173 (5)
N1	0.5786 (2)	0.1557 (14)	0.6749 (3)	0.0362 (9)	0.827 (5)
N1A	0.707 (4)	0.494 (19)	0.611 (3)	0.0366 (12)	0.173 (5)
C1	0.64614 (15)	0.4012 (7)	0.5486 (2)	0.0309 (6)	
C2	0.64506 (14)	0.4585 (7)	0.45317 (18)	0.0258 (6)	
C3	0.70969 (14)	0.6130 (7)	0.4138 (2)	0.0286 (6)	
H3	0.7523	0.6791	0.4492	0.034*	
C4	0.71138 (15)	0.6687 (7)	0.3247 (2)	0.0320 (6)	
H4	0.7554	0.7696	0.2979	0.038*	
C5	0.64841 (16)	0.5767 (7)	0.27380 (19)	0.0312 (6)	
H5	0.6495	0.6167	0.2120	0.037*	
C6	0.58366 (15)	0.4269 (7)	0.31197 (19)	0.0281 (6)	
H6	0.5407	0.3701	0.2762	0.034*	
C7	0.58119 (14)	0.3592 (7)	0.40243 (18)	0.0244 (6)	
C8	0.51377 (13)	0.1929 (7)	0.44476 (18)	0.0257 (6)	
C9	0.45080 (15)	0.0821 (7)	0.3952 (2)	0.0292 (6)	
H9	0.4511	0.1139	0.3330	0.035*	
C10	0.38781 (15)	−0.0737 (8)	0.4350 (2)	0.0325 (7)	
H10	0.3458	−0.1498	0.4000	0.039*	
C11	0.38582 (16)	−0.1190 (8)	0.5257 (2)	0.0356 (7)	
H11	0.3421	−0.2200	0.5531	0.043*	
C12	0.44774 (15)	−0.0164 (8)	0.5756 (2)	0.0328 (7)	
H12	0.4466	−0.0500	0.6377	0.039*	
C13	0.51259 (15)	0.1372 (7)	0.53659 (19)	0.0286 (6)	
C14	0.57893 (17)	0.2359 (7)	0.59059 (19)	0.0319 (6)	

### Atomic displacement parameters (Å^2^)

**Table:**

	*U*^11^	*U*^22^	*U*^33^	*U*^12^	*U*^13^	*U*^23^
O1	0.0292 (16)	0.0496 (13)	0.031 (3)	−0.0041 (12)	−0.007 (2)	−0.003 (3)
O1A	0.023 (2)	0.050 (2)	0.0349 (16)	−0.001 (2)	−0.0061 (19)	0.0011 (15)
O2	0.0401 (14)	0.073 (2)	0.0368 (15)	−0.0153 (15)	−0.0084 (11)	0.0053 (14)
O2A	0.0401 (14)	0.073 (2)	0.0368 (15)	−0.0153 (15)	−0.0084 (11)	0.0053 (14)
N1	0.023 (2)	0.050 (2)	0.0349 (16)	−0.001 (2)	−0.0061 (19)	0.0011 (15)
N1A	0.0292 (16)	0.0496 (13)	0.031 (3)	−0.0041 (12)	−0.007 (2)	−0.003 (3)
C1	0.0330 (15)	0.0237 (13)	0.0359 (15)	0.0060 (11)	0.0007 (12)	−0.0048 (12)
C2	0.0236 (12)	0.0177 (12)	0.0360 (15)	0.0036 (10)	0.0021 (10)	−0.0013 (12)
C3	0.0228 (13)	0.0207 (13)	0.0424 (17)	0.0024 (9)	0.0010 (11)	−0.0014 (12)
C4	0.0256 (13)	0.0234 (13)	0.0470 (17)	0.0010 (10)	0.0088 (13)	0.0020 (12)
C5	0.0320 (13)	0.0253 (14)	0.0364 (16)	0.0055 (11)	0.0077 (12)	0.0058 (12)
C6	0.0253 (13)	0.0230 (13)	0.0360 (15)	0.0049 (10)	−0.0024 (11)	−0.0015 (12)
C7	0.0215 (13)	0.0152 (12)	0.0364 (15)	0.0056 (9)	0.0022 (10)	−0.0026 (11)
C8	0.0222 (12)	0.0152 (12)	0.0397 (16)	0.0066 (10)	0.0035 (11)	−0.0020 (11)
C9	0.0253 (12)	0.0214 (13)	0.0408 (16)	0.0050 (10)	0.0015 (12)	−0.0016 (12)
C10	0.0214 (12)	0.0233 (13)	0.0530 (19)	0.0023 (10)	0.0000 (12)	−0.0045 (12)
C11	0.0272 (14)	0.0235 (14)	0.056 (2)	0.0049 (11)	0.0128 (12)	0.0020 (13)
C12	0.0334 (14)	0.0235 (13)	0.0415 (17)	0.0033 (11)	0.0090 (12)	0.0004 (12)
C13	0.0296 (14)	0.0192 (12)	0.0370 (15)	0.0071 (10)	0.0074 (11)	−0.0014 (12)
C14	0.0384 (15)	0.0259 (14)	0.0315 (15)	0.0033 (12)	0.0033 (11)	−0.0010 (12)

### Geometric parameters (Å, º)

**Table:**

O1—C1	1.233 (8)	C5—C6	1.391 (4)
O1A—C14	1.25 (2)	C5—H5	0.9500
O1A—H1A	0.8400	C6—C7	1.396 (4)
O2—N1	1.371 (5)	C6—H6	0.9500
O2—H2	0.8400	C7—C8	1.481 (3)
O2A—N1A	1.11 (5)	C8—C9	1.396 (4)
N1—C14	1.314 (6)	C8—C13	1.409 (4)
N1A—C1	1.46 (5)	C9—C10	1.386 (4)
C1—C2	1.464 (4)	C9—H9	0.9500
C1—C14	1.474 (4)	C10—C11	1.386 (4)
C2—C3	1.404 (4)	C10—H10	0.9500
C2—C7	1.406 (4)	C11—C12	1.376 (4)
C3—C4	1.369 (4)	C11—H11	0.9500
C3—H3	0.9500	C12—C13	1.403 (4)
C4—C5	1.387 (4)	C12—H12	0.9500
C4—H4	0.9500	C13—C14	1.467 (4)
			
C14—O1A—H1A	109.5	C2—C7—C8	120.4 (2)
C14—N1—O2	119.9 (4)	C9—C8—C13	118.4 (3)
O2A—N1A—C1	112 (5)	C9—C8—C7	121.3 (2)
O1—C1—C2	119.7 (4)	C13—C8—C7	120.3 (2)
N1A—C1—C2	128 (2)	C10—C9—C8	121.1 (3)
O1—C1—C14	121.6 (4)	C10—C9—H9	119.4
N1A—C1—C14	114 (2)	C8—C9—H9	119.4
C2—C1—C14	118.7 (2)	C11—C10—C9	120.3 (3)
C3—C2—C7	121.1 (2)	C11—C10—H10	119.8
C3—C2—C1	118.1 (2)	C9—C10—H10	119.8
C7—C2—C1	120.8 (2)	C12—C11—C10	119.4 (3)
C4—C3—C2	120.1 (3)	C12—C11—H11	120.3
C4—C3—H3	120.0	C10—C11—H11	120.3
C2—C3—H3	120.0	C11—C12—C13	121.2 (3)
C3—C4—C5	119.6 (3)	C11—C12—H12	119.4
C3—C4—H4	120.2	C13—C12—H12	119.4
C5—C4—H4	120.2	C12—C13—C8	119.4 (3)
C4—C5—C6	121.0 (3)	C12—C13—C14	120.4 (3)
C4—C5—H5	119.5	C8—C13—C14	120.2 (2)
C6—C5—H5	119.5	O1A—C14—C13	103.3 (9)
C5—C6—C7	120.6 (3)	N1—C14—C13	118.8 (3)
C5—C6—H6	119.7	O1A—C14—C1	135.9 (10)
C7—C6—H6	119.7	N1—C14—C1	121.4 (3)
C6—C7—C2	117.7 (2)	C13—C14—C1	119.7 (2)
C6—C7—C8	121.9 (2)		

### Hydrogen-bond geometry (Å, º)

**Table:**

*D*—H···*A*	*D*—H	H···*A*	*D*···*A*	*D*—H···*A*
O2—H2···O1	0.84	1.76	2.499 (8)	146
O1*A*—H1*A*···O2*A*	0.84	1.93	2.54 (2)	128
